# Characterization and Identification of MicroRNA Core Promoters in Four Model Species

**DOI:** 10.1371/journal.pcbi.0030037

**Published:** 2007-03-09

**Authors:** Xuefeng Zhou, Jianhua Ruan, Guandong Wang, Weixiong Zhang

**Affiliations:** 1 Department of Computer Science and Engineering, Washington University in Saint Louis, Saint Louis, Missouri, United States of America; 2 Department of Genetics, Washington University in Saint Louis, Saint Louis, Missouri, United States of America; Whitehead Institute, United States of America

## Abstract

MicroRNAs are short, noncoding RNAs that play important roles in post-transcriptional gene regulation. Although many functions of microRNAs in plants and animals have been revealed in recent years, the transcriptional mechanism of microRNA genes is not well-understood. To elucidate the transcriptional regulation of microRNA genes, we study and characterize, in a genome scale, the promoters of intergenic microRNA genes in *Caenorhabditis elegans, Homo sapiens, Arabidopsis thaliana,* and Oryza sativa. We show that most known microRNA genes in these four species have the same type of promoters as protein-coding genes have. To further characterize the promoters of microRNA genes, we developed a novel promoter prediction method, called *common query voting* (CoVote), which is more effective than available promoter prediction methods. Using this new method, we identify putative core promoters of most known microRNA genes in the four model species. Moreover, we characterize the promoters of microRNA genes in these four species. We discover many significant, characteristic sequence motifs in these core promoters, several of which match or resemble the known *cis*-acting elements for transcription initiation. Among these motifs, some are conserved across different species while some are specific to microRNA genes of individual species.

## Author Summary

MicroRNAs are a class of short RNA sequences that have many regulatory functions in complex organisms such as plants and animals. However, our knowledge of the transcriptional mechanisms of microRNA genes is limited. Here, we analyze the upstream sequences of known microRNA genes in four model species, i.e., *C. elegans, H. sapiens, A. thaliana,* and *O. sativa,* and compare them with the promoter sequences of protein-coding genes and other classes of RNA genes. This analysis provides genome-wide evidence that microRNA genes have the same type of promoter sequences as protein-coding genes, and therefore are likely transcribed by RNA polymerase II (pol II). Second, we present a novel computational method for promoter prediction, which is then applied to locate the core promoters of known microRNA genes in the four model species. Furthermore, we present an analysis of short DNA motifs that appear frequently in the predicted promoters of microRNA genes, and report several interesting motifs that may have some functional meanings. These results are important for understanding the initiation and regulation of microRNA gene transcription.

## Introduction

MicroRNAs are endogenous single-stranded RNAs ranging from 19–25 nt in length. They are generated from long precursors, which fold into hairpin structures, and are known to repress post-transcriptional gene expression in both animals and plants [1,2]. The two well-understood microRNAs, *lin-4* and *let-7,* were discovered in the 1990s, and proved to regulate developmental timing in C. elegans by repressing the translation of a family of key mRNAs [3–5]. Since then, several hundred microRNAs have been identified in viruses, plants, and animals, and their important post-transcriptional regulatory functions have been discovered.

The biogenesis of microRNAs is complex. Most microRNAs are encoded in their own genes situated in intergenic regions or located on the antisense strands of annotated genes [6–8]. The intergenic microRNA genes are believed to be transcribed independently and to form a new gene family, whereas the intronic ones and the ones interspersed with mobile elements Alu in the human genome can be transcribed with their host genes [9,10]. Our knowledge of post-transcriptional processing of microRNAs has greatly expanded in recent years through various studies [11–14]. However, we have limited understanding of the transcription of microRNA genes, which is the first, and an important, step of microRNA biogenesis. In this study, we are interested in the known microRNA genes that contain their own transcriptional units.

Many pieces of evidence have indirectly suggested that microRNA genes are *class-II* genes (i.e., genes transcribed by RNA polymerase II (pol II)). For instance, primary transcripts of some microRNA genes contain poly(A) tails, or the cap structure [15,16]. Expressions of some microRNA genes are regulated by enhancers [17,18] or hormones [19]. Lee et al. reported the first direct evidence from an experiment on a single polycistronic microRNA gene, *mir-23a∼27a∼24–2,* showing that it can be transcribed by pol II [20]. They also determined the promoter and terminator regions of this gene. However, their results, especially those on the promoter of *mir-23a∼27a∼24–2,* do not match very well with our knowledge of pol II promoters. Specifically, the promoter of *mir-23a∼27a∼24–2* appears to lack the known common promoter elements required for initiating transcription, such as the TATA-box, initiator element, downstream promoter element (DPE), TFIIB recognition element (BRE) [20], or the proximal sequence element (PSE). Additionally, they also found that a large portion of a given pri-microRNA (the primary transcript of an microRNA gene) does not contain a 5′ cap or a poly(A) tail [20]. Another piece of experimental evidence was from a M. musculus polycistronic microRNA gene, *mmu-mir-290∼291∼292∼293∼294∼295*. Houbaviy et al. found a canonical TATA-box, located at −35, of capped and polyadenylated pri-microRNA of this gene, and showed that this upstream region was also conserved in a H. sapiens homologous gene, *hsamir-371∼372∼373* [21]. Furthermore, Xie et al. identified the promoters of 52 A. thaliana microRNA genes, and showed that most of them have TATA-boxes in their core promoters [22].

All these results are fundamentally important; they have provided direct evidence that a microRNA gene can be transcribed by pol II. However, a few critical questions remain unanswered. One of them is whether *all* known microRNA genes of different species are *class-II* genes. Although more than 50 A. thaliana microRNA genes have been shown to be transcribed by pol II, our knowledge of the transcription of microRNA genes in animals is still limited. We consider this important issue through a genome-wide computational analysis on four model species, *C. elegans, H. sapiens, A. thaliana,* and O. sativa. Our overall strategy is based on the following perspective on transcriptional regulation. *Class-II* genes and *class-III* genes (genes transcribed by RNA polymerase III) must have distinctive features in their promoter regions, including transcription factor binding motifs, to recruit the right transcriptional machineries to initiate their transcription. Based on this perspective and supported in part by the results in [20–22], we first assume that the core promoters of intergenic microRNA genes share common sequence features with the core promoters of the known *class-II* or *class-III* genes. We then build computational models to separate the core promoters of *class-II* and *class-III* genes as well as random sequences. Using these models, we test all known intergenic microRNA genes in the four species to determine what types of promoters they have. We subsequently answer the question: which RNA polymerase is responsible for the transcription of these microRNA genes?

The promoter of a gene is a crucial control region for its transcription initiation [23,24]. To understand the mechanism and conditions of the activation of microRNA genes, it is required to locate their core promoter regions. One practical way to identify core promoters of microRNA genes is to first apply a promoter prediction method to predict their core promoters, and then to verify the predictions by wet lab experiments. Developing the promoter identification algorithm is a very challenging problem. Although computational methods have been developed for predicting core promoters of protein-coding genes, their performances are far from satisfactory. The main reason is that our understanding of the transcription process is incomplete. The situation with microRNA genes is even worse. All existing promoter prediction methods for protein-coding genes may not be suitable for microRNA genes, since they were not built based on the core promoters of microRNA genes. Furthermore, the promoters of most microRNA genes in all species remain undefined. For *H. sapiens,* only the promoters of two microRNA genes, *hsa-mir23a∼27a∼24–2* [20] and *hsa-mir-371∼372∼373* [21], have been identified so far. The promoter of *hsa-mir-23a∼27a∼24–2* has been located by biological experiments [20], while the promoter of *hsa-mir-371∼372∼373* [21] has been identified by a comparative genomic analysis. The 52 microRNA genes in A. thaliana studied in [22] are not sufficient to build a good predictive model.

Core promoter regions contain essential components for the regulation of gene transcription [23,24]. The basal transcription machinery, comprising the multisubunit RNA polymerase and several auxiliary factors, is thought to interact directly with core promoter elements [23,24]. Thus, revealing functional regulatory binding sites in promoter regions is important for determining promoter structures and characterizing transcriptional regulation. However, core promoter elements are highly variable, requiring sophisticated techniques for their detection. Discovering key *cis*-elements of microRNA genes is more difficult, since our knowledge about the transcription of this novel family of genes is limited. Lee et al. located the promoter of *mir-23a∼27a∼24–2*; however, none of the canonical promoter elements were discovered in this promoter [20]. TATA-box was found in *mmu-mir-290∼291∼292∼293∼294∼295* [21]. However, the deletion of this putative TATA-containing promoter region had almost no effect on the expression level of *mir292* and the precursor to *mir292* in transfected cell lines [21]. Ohler et al. scanned the 1,000-bp upstream sequences of *Drosophila* microRNA genes for known promoter motifs, but did not detect a consistent preference for any known motifs that are enriched in protein-coding genes [25].

In this study, we propose a novel promoter prediction approach, CoVote (common query voting), for predicting microRNA core promoters. Using CoVote, we investigate core promoter regions of microRNA genes in *C. elegans, H. sapiens, A. thaliana,* and O. sativa, and further analyze sequence motifs in the putative core promoters that may be involved in the transcription of microRNA genes. Our objectives are to (1) identify characteristic motifs in core promoters of known microRNA genes in these four species, and (2) compare the potential promoter structure of microRNA genes in different species. We examine the presence and distribution of conserved motifs in these species, and also investigate species-specific motifs.

## Materials and Methods

### Datasets

Two discriminative models were built and used in our study. The first model (the three-class model, discussed in Discriminative Models of Pol II and Pol III Promoters) is for discriminating the promoters of genes transcribed by RNA polymerases II (pol II promoters) and the promoters of genes transcribed by RNA polymerases III (pol III promoters), as well as random sequences. To build this model, we prepared training sequences of three different types: known pol II core promoter sequences, known pol III core promoter sequences, and random sequences. The numbers of these sequences are listed in Table 1. The second model is for identifying putative promoters of microRNA genes. This model only needs to separate pol II promoter sequences and random sequences (see The CoVote Algorithm for Locating Core Promoter Regions of MicroRNA Genes). Therefore, we only used these two types of sequences as training data.

**Table 1:**
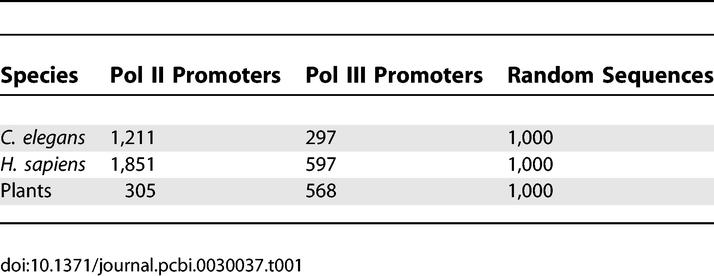
The Numbers of Training Sequences for Building Discriminative Models

The pol II sequences were downloaded from the Web as of March 2005. The C. elegans core pol II promoters were retrieved from C. elegans promoter database (CEPDB) (http://rulai.cshl.edu/cgi-bin/CEPDB/home.cgi). The H. sapiens pol II promoters were downloaded from the Eukaryotic Promoter Database (EPD) (http://www.epd.isb-sib.ch/seq_download.html). The plant core pol II promoters were obtained from Plant Promoter Database (PlantProm) (http://mendel.cs.rhul.ac.uk/mendel.php?topic=plantprom). All these sequences are 250 bp long and cover the regions from −200 bp to +50 bp with respect to the corresponding transcription start sites.

The known core promoter sequences of A. thaliana and O. sativa are not sufficient to build a discriminative model. As shown in Table 2, we thus included the pol II promoter sequences from 44 dicotyledonous and seven monocotyledonous plants in our study. Both the discriminative model for pol II and pol III promoters and the promoter prediction model trained with these sequences were applied to A. thaliana and O. sativa.

**Table 2:**
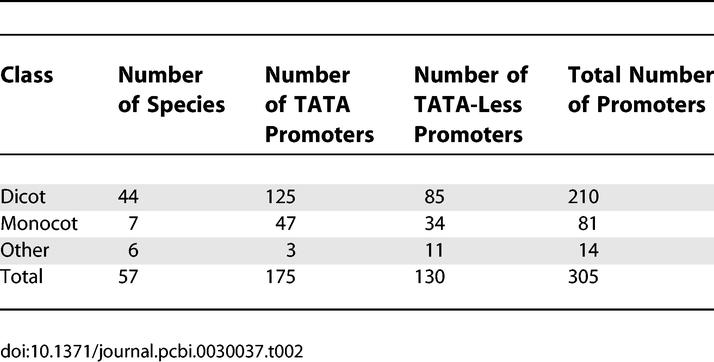
The Numbers of Pol II Promoter Sequences from Different Species Included in the Training Set for Plant Models

For each species, the pol III promoter sequences that we used included the promoter sequences of tRNAs, U6 snRNAs, 7SL RNAs, and 7SK RNAs (Table 3). The promoter of each tRNA covered the complete coding region of the tRNA and its upstream sequence with a total length of 250 bp. The promoters of U6 snRNA, 7SL RNA, and 7SK RNA included 200-bp upstream sequences and 50-bp downstream sequences, relative to their transcription start sites (TSSs). The sequences of these ncRNAs were downloaded from the ncRNA database (http://noncode.bioinfo.org.cn/showclass.php?class=snRNA).

**Table 3:**
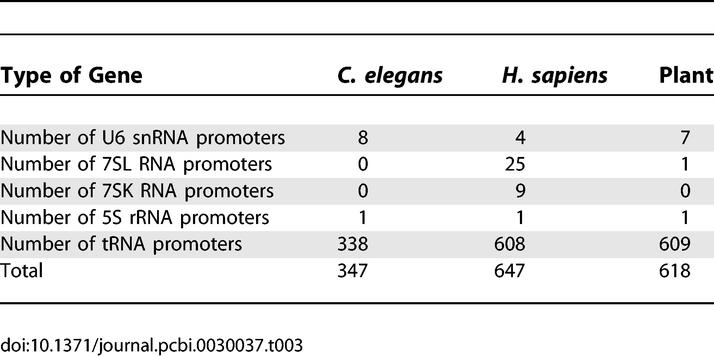
The Numbers of Different *class-III* Promoters in Pol III Training and Test Sets

Since availability of known pol III promoters is limited, we randomly chose 50 pol III promoter sequences from *C. elegans, H. sapiens,* and plants, respectively, as independent test sets for corresponding discriminative models.

We generated 1,000 random sequences of 250 bp length to represent intergenic sequences other than pol II and pol III core promoter sequences. For each species, we used the nucleotide composition of intergenic regions of its genome to generate these sequences. We did not use intergenic sequences from a genome for this purpose because it is difficult to ensure that intergenic sequences do not overlap with real promoter regions.

Three independent test sets for each species studied were used to validate the three-class discriminative model. The first set included 1,000-bp upstream sequences of 1,000 randomly chosen coding genes. These sequences were obtained from RSA Tools (http://rsat.ulb.ac.be/rsat/).The second set contained the 50 pol III promoters not used in training. The last set of sequences included 1,000 randomly generated sequences of 2,000 bp length. We applied the nucleotide composition of pol II and pol III promoter sequences to generate 500 sequences, respectively, for each species.

Two independent sets were also prepared to validate the promoter prediction model. The first set includes 4,189 H. sapiens pol II promoters, downloaded from the Database of Transcriptional Start Sites (DBTSS) (http://dbtss.hgc.jp/samp_home.html). The second set contained 4,000 sequences randomly chosen from H. sapiens protein coding regions.

For each species studied, the upstream sequences of pre-microRNAs (hairpin precursors) of the intergenic microRNA genes were obtained as follows. First, when a pre-microRNA and its upstream gene were unidirectional (same direction), if the distance between them was longer than 2,400 bp, the 2,000-bp sequence upstream of the pre-microRNA was retrieved; otherwise, the sequence between 400 bp downstream of the upstream gene and the precursor was used. Second, when a pre-microRNA and its upstream gene were convergent (opposite directions), if the distance between them was longer than 4,000 bp, the 2,000-bp sequence upstream of the precursor was obtained; otherwise, the sequence from the precursor and the middle point between the upstream gene and the precursor was retrieved. Some C. elegans and H. sapiens microRNA genes are polycistronic, in which case only upstream sequences of the 5′ pre-microRNAs were considered in our study. In addition to intronic microRNA genes, the ones in human that are interspersed and transcribed with Alu elements were excluded from our analysis.

### Feature (Sequence Motif) Extraction

Our overall approach depends on building accurate discriminative models of transcriptional regulation, which in turn rely on sequence features. We may simply use all possible *k*-mers, with reasonable values of *k,* as such features. However, not all *k*-mers have the same amount of information, and the number of *k*-mers increases exponentially with *k*. The key then is to find a sufficient number of statistically overrepresented motifs in the sequences of interest.

We used the WordSpy algorithm developed by Wang et al. [26,27] to find significant motifs, for several reasons. Statistical modeling and word counting methods have been integrated in WordSpy; it is able to build a dictionary of a large number of statistically significant motifs. WordSpy adopts a strategy of steganalysis, which is a technique for discovering hidden patterns and information from a medium such as strings, so that it does not have to rely on additional background sequences and is still able to find motifs of nearly exact lengths.

### Discriminative Models of Pol II and Pol III Promoters

It is believed that Pol II and Pol III transcribe different types of genes whose promoters are intrinsically different from each other and from other genomic sequences [23]. Therefore, it is viable to assume that the core promoters of these two classes of genes have discriminative sequence features that separate them from each other and from the other genomic sequences. Consequently, a discriminative model can be built using the known promoters of these two types of genes, and be used to determine if query sequences are pol II promoters, pol III promoters, or other intergenic sequences.

Specifically, we built a three-class discriminative model, or classifier, to distinguish pol II promoters, pol III promoters, and random intergenic sequences for each of the four species that we studied, i.e., *C. elegans, H. sapiens, A. thaliana,* and O. sativa. We extracted statistically overrepresented sequence motifs of 5–10-bp length from each training set separately, using the WordSpy motif-finding algorithm [26]. With these sequence motifs as features, we represented each promoter sequence as a vector, where an entry in the vector was the number of occurrences of a motif in the sequence. We then built two classifiers for each species, one using a decision tree [28], the other using a support vector machine (SVM) [29] to separate the three types of sequences. We adopted these two well-studied classification methods to ensure that our analysis of microRNA genes is not skewed by the computational methods used.

We applied the SVM implementation in the WEKA software package [30] under its default setting. We tested linear, polynomial, and radial kernels [29]. Although the cross-validation accuracies of the polynomial and radial kernels were slightly better than that of the linear kernel, we used the linear kernel due to its simplicity. For the decision tree learning, we applied the J48 program in WEKA [30], which is an implementation of the well-known C4.5 algorithm [28]. To prevent overfitting, we required each leaf node to have at least five sequences.

The accuracies of the discriminative models were estimated using a 10-fold cross-validation. In this process, a training set was randomly partitioned into ten roughly equal-sized subsets. Each subset was then used in turn as a test set to estimate the prediction quality of the model built with the other nine subsets. The average quality of these tests was the final accuracy measure. To measure prediction quality, we calculated recall, precision, and overall accuracy for each type of sequence. The recall for pol II promoters (respectively, III) was defined as the ratio of the number of correctly predicted pol II (respectively, III) sequences versus the total number of pol II (respectively, III) sequences tested. The precision was defined as the ratio of the number of correctly predicted pol II (respectively, III) sequences versus the total number of predicted pol II (respectively, III) sequences. The overall accuracy was defined as the number of correctly predicted sequences versus the total number of sequences tested.

When we applied the discriminative models to predict the type of promoter that a query gene may have, the upstream sequence of the query gene was fragmented using a sliding window of 250 bp, with an increment of 50 bp. Each segment was then tested by the discriminative models separately. The experimental results were organized in five categories. The first category contained the upstream sequences in which at least one of the 250-bp segments was classified as pol II promoter and none of the rest were predicted as pol III promoter. This class, called *definitive pol II class,* provided the definitive evidence for *class-II* genes. The second category had the sequences in which some of the segments were classified as pol II and some as pol III promoters, but there were more pol II segments than pol III segments. We called this category *possible pol II class,* since we simply classified a sequence to be a pol II promoter based on the majority prediction for its segments. The next category, called *possible pol III class,* was similar to the second, but the number of pol III segments was greater than the number of pol II segments. The fourth category, called *definitive pol III class,* had sequences in which at least one segment was a pol III promoter but none of the rest was predicted as a pol II promoter. The last category, called *random class,* contained sequences with all segments classified as random promoters.

### The CoVote Algorithm for Locating Core Promoter Regions of MicroRNA Genes

Our method, which we called *common query voting*, shorthanded as CoVote, is based on the following understanding of the promoters of the microRNA gene. MicroRNA genes have the same type of promoters as other *class-II* genes, as shown in this paper and in [20–22]. Therefore, there must be characteristic sequence features in the core promoters of microRNA genes with respect to random sequences that have the same nucleotide compositions of intergenic sequences. Moreover, compared with other upstream regions, core promoters should be the most similar upstream regions among most, if not all, microRNA genes. Although the promoters of microRNA genes have some similar, or even the same, features as promoters of the known *class-II* genes, they may have their own unique features that have not been discovered. Compared with many existing promoter prediction methods, CoVote not only takes into account the features that the training instances have, but also captures potential common features in many query instances. The CoVote algorithm runs as follows.

#### Model training step.

Train a two-class decision tree model with some known pol II promoters as positive examples and some randomly generated sequences as negative training examples, in a way similar to the three-class discriminative models described in the section Discriminative Models of Pol II and Pol III Promoters.

#### Classification step.

Apply the two-class model to the upstream sequences of microRNA genes, fragmented into overlapping 250-bp segments as described previously in Discriminative Models of Pol II and Pol III Promoters. Each segment is predicted to be either a pol II promoter or a random sequence by the tree at one of its leaf nodes. The classification of a segment corresponds to following a path from the root to a leaf node in the tree, and the nodes on the path represent the sequence motifs used. Therefore, the decision tree model provides a mechanism for identifying the segments that are most likely to belong to the same core promoter class using the same set of sequence motifs.

#### Scoring step.

Each leaf node is assigned a weight equal to the number of microRNA genes that have at least one upstream segment classified to be a pol II promoter at that leaf node. Then, the score of each upstream segment that has been predicted to be a pol II promoter is the weight of the leaf node at which it is classified. This weighting scheme explicitly takes into account the similarities among the putative promoters of microRNA genes themselves. The weight of a leaf node reflects how many upstream sequences follow the rule specified by the path from the root node to this leaf node. Since the score of a segment can be viewed as a vote of other similar segments, we name our method *common query voting* (CoVote).

#### Putative promoter identification step.

For each microRNA gene, consecutive segments of nonzero scores in its upstream sequence are combined. The score of the combined subsequence is the sum of the scores of these consecutive segments. All these combined subsequences are then taken to be the putative core promoter regions of the microRNA gene according to a user-specified cutoff score. Some microRNA genes may be predicted to have multiple putative promoter regions.

### Motif Analysis

We applied the WordSpy algorithm to identify significant motifs from putative core microRNA promoters. Furthermore, in addition to WordSpy, we also applied the popular MEME algorithm [31] with its default parameters to find 20 top-ranking degenerate motifs for each species considered.

It is critical to ensure that the motifs from putative core microRNA promoters are indeed specific to promoters. For this purpose, we used a whole-genome Monte Carlo simulation to measure the specificity and significance of a motif in the putative promoters, which we call *target set,* with respect to a set of different sequences, which we call *reference set*. A reference set can be drawn from other regions of a genome. For example, in this research, we randomly chose reference sets from open reading frames (ORFs) and other genome regions. Given a motif of interest, we computed its *Z*-score with respect to other regions of the genome as follows. We first obtained the average number of occurrences per target sequence for the motif, denoted as *Nt*. We then randomly generated a large number of reference sets and computed the average number of occurrences of the motif, *Nr*, and its standard deviation, *σr*, over the reference sets. The *Z*-score was then calculated as *Z* = (*Nt/Nr*) *= σr*. Here, we set the size of a reference set to be the same as that of the target set. Therefore, all the reference sets can be considered as independently and identically distributed, and follow a normal distribution when the number of samples is large. Consequently, the *Z*-score simply measures the normalized difference between the average occurrence of the motif in the target set and the sample mean in the reference sets. For example, if the *Z*-score is 2, the specificity of the motif to the target set is two times the standard deviation to the example mean of the reference sets.

## Results/Discussion

### Accuracy of the Three-Class Discriminative Models

We evaluated the quality of the three-class discriminative models in terms of recall, precision, and accuracy (see Discriminative Models of Pol II and Pol III Promoters). Table 4 lists the 10-fold cross-validation results of the SVM and decision tree–based classifiers. The results show that these discriminative models are fairly accurate, with the minimum accuracy greater than 96% for the SVM models and greater than 87% for the decision tree models. The SVM models are marginally better than the decision-tree models.

**Table 4:**
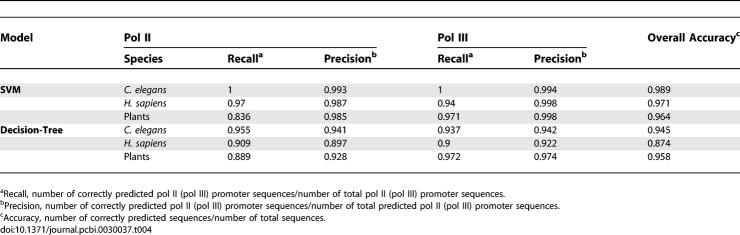
Results of 10-Fold Cross-Validations of SVM and Decision-Tree Models

To further examine the accuracy of the models, we assessed the error rates by control experiments on independent test sets (see Datasets). The decision-tree models have comparable but slightly worse classification accuracies than the SVM models, so the results are omitted. For each of the three SVM-based models, their accuracies were examined on three independent test sets.

The first set includes promoter sequences of randomly chosen protein coding genes. Since the protein coding genes contain pol II promoters, the percentage of protein coding genes predicted to have pol III promoters will reflect the error rates of these discriminative models. The error rates of the SVM models are shown in Table 5. Among 1,000 coding genes, only a handful of them were predicted to have *possible pol III* or *definitive pol III* promoters (i.e., eight C. elegans genes, 25 H. sapiens genes, and 31 plant genes).

**Table 5:**
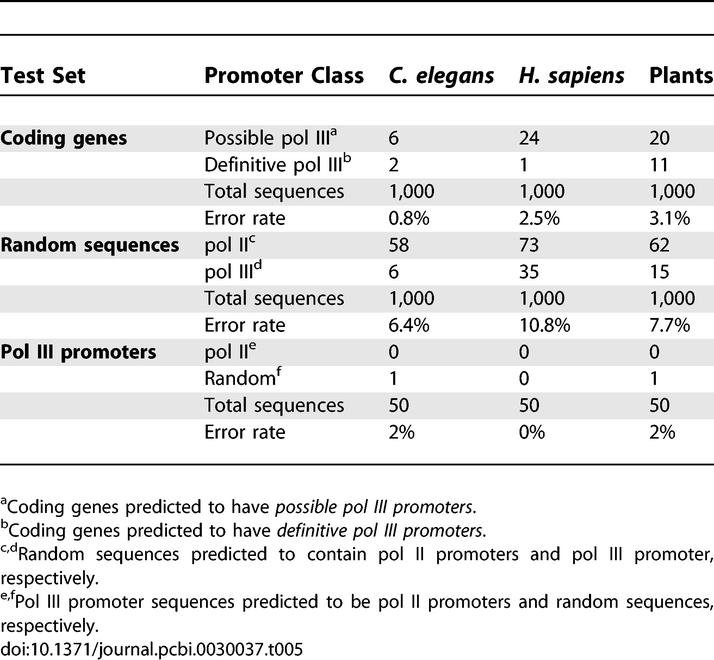
Error Rates of SVM Models on Independent Test Sets

The second independent set contains 1,000 random sequences of 2,000 bp length. Half of these sequences have the same nucleotide composition as pol II promoter sequences, while the other half have the same nucleotide composition as pol III promoter sequences. We used randomly generated intergenic sequences instead of real intergenic sequences, since it is difficult to ensure that the intergenic sequences do not to overlap with real promoter regions. As shown in Table 5, the error rates of the discriminative models on randomly generated sequences for *C. elegans, H. sapiens,* and plants are 6.4%, 10.8%, and 7.7%, respectively.

Moreover, since experimentally verified pol III promoters are very limited, we saved 50 pol III promoter sequences from *C. elegans, H. sapiens,* and plants, respectively, as independent test sets. As shown in Table 5, for the discriminative models on pol III promoters from *C. elegans, H. sapiens,* and plants, the error rates are 2%, 0%, and 2%, respectively.

Based on the cross-validation and these three independent tests, we can conclude that (1) pol II and pol III promoters can be separated from each other and are also distinguishable from random intergenic sequences, and (2) the quality of the discriminative models that we developed is sufficiently high.

### Most MicroRNA Genes Have Pol II Promoters

To determine the promoter types of the known intergenic microRNA genes of the four model species, we conducted two experiments using the three-class discriminative models that we developed. We considered separately the precursors (pre-microRNAs) and primary transcripts (pri-microRNAs) of known microRNAs. We analyzed upstream sequences up to 2,000 bp of these transcripts. As described in the section Discriminative Models of Pol II and Pol III Promoters, these upstream sequences were fragmented using a sliding window of 250 bp, with an increment of 50 bp. Each segment was then tested by the discriminative models separately, and the experimental results were organized into five categories: *definitive pol II class, possible pol II class, possible pol III class, definitive pol III class,* and random class, as discussed in Discriminative Models of Pol II and Pol III Promoters.

Table 6 shows the results on the four species using the SVM models. The results from the decision tree models were similar. We tested 73 *C. elegans,* 109 *H. sapiens,* 112 *A. thaliana,* and 114 O. sativa pre-microRNAs that are in intergenic regions according to the genome annotation as of March 2005. Among them, 67 (91.8%) *C. elegans,* 81 (74.3%) *H. sapiens,* 81 (72.3%) *A. thaliana,* and 92 (80.7%) O. sativa microRNAs have *definitive pol II* class promoters. These results suggest that most microRNA genes in the four species have the same promoters as protein coding genes. However, six (8.2%), 24 (22%), 17 (15.2%), and 12 (10.5%) microRNAs of these species have *possible pol II* class promoters, respectively. One *H. sapiens,* three *A. thaliana,* and one O. sativa microRNA genes were predicted to have *possible pol III* promoters. In the upstream regions of these microRNA genes, some segments were predicted to be pol II promoters while some were predicted to be pol III promoters. Combining the microRNAs in these two categories, 73 (100%) *C. elegans,* 105 (96.3%) *H. sapiens,* 98 (87.5%) *A. thaliana,* and 104 (91.2%) O. sativa microRNA genes have pol II promoters. Importantly, none of the microRNA genes were predicted to have a *definitive pol III* promoter, and only one *H. sapiens,* three *A. thaliana,* and one O. sativa microRNA genes were predicted to have *possible pol III* promoters.

**Table 6:**
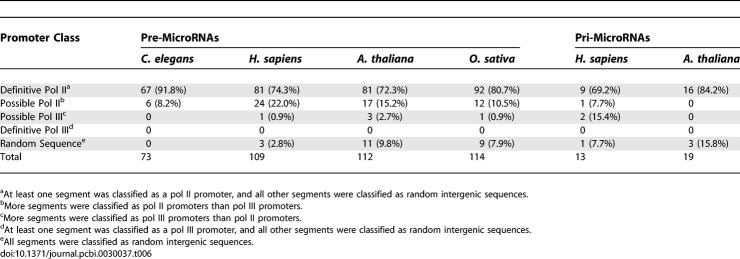
Classification Results of MicroRNA Genes Using the Known Pre-MicroRNAs and Pri-MicroRNA

Similar results, shown in Table 6, were obtained on H. sapiens and A. thaliana pri-microRNAs. We expected the results based on pri-microRNAs to be more definitive than those from pre-microRNAs. However, we were only able to find 13 pri-microRNAs for H. sapiens and 19 pri-microRNAs for A. thaliana. It is difficult to draw a meaningful conclusion based on such limited samples. Nevertheless, as shown in Table 6, nine out of 13 (69.2%) H. sapiens microRNAs and 16 out of 19 (84.2%) A. thaliana microRNAs were predicted to have definitive pol II promoters.

These results provided *genome-wide* evidence that most microRNA genes are *class-II* genes and have pol II promoters. This is consistent with the previous study on a polycistronic H. sapiens microRNA gene, *mir-23a∼27a∼24–2* [20], and the report on some A. thaliana microRNA genes [22].

### Core Promoters of MicroRNA Genes

In this research, we developed a novel computational, sequence-centric method, CoVote, for identifying the core promoter regions of microRNA genes, as described in the section The CoVote Algorithm for Locating Core Promoter Regions of MicroRNA Genes. Using CoVote, we predicted putative core promoters for most known microRNA genes of the four species. Specifically, we predicted promoters for all of the 73 tested C. elegans microRNA genes, 107 (98.2%) of 109 tested H. sapiens microRNA genes, 95 (84.8%) of 112 tested A. thaliana microRNA genes, and all of the 114 tested O. sativa microRNA genes. Among the microRNA genes whose promoters were identified by CoVote, some were predicted to contain multiple core promoter regions. Figure 1 shows the distributions of the positions of putative promoters with respect to corresponding microRNA foldbacks (the first foldbacks of polycistronic microRNA genes). In short, 70 (95.9%) of 73 C. elegans microRNA genes, 100 (93.5%) of the 107 H. sapiens microRNA genes, 80 (84.2%) of 95 A. thaliana microRNA genes, and 109 of 114 (96.6%) O. sativa microRNA genes have putative promoters within 500 bp of upstream regions. This distribution pattern may imply that real core promoters of most microRNA genes are close to pre-microRNA hairpins.

**Figure 1:**
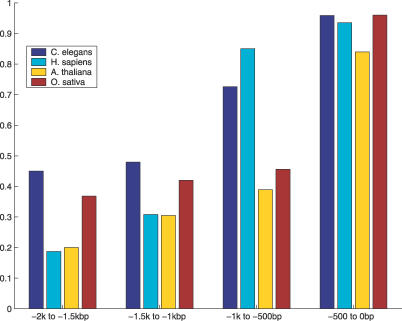
The Distribution of the Distances between Putative Promoters and MicroRNA Hairpins The horizontal axis shows the positions of putative promoters with respect to the corresponding microRNA hairpins and the vertical axis shows the percentage of microRNA genes that have putative promoters at the specified positions.

Recently, Xie et al. experimentally identified 65 core promoters of 52 A. thaliana microRNA genes (multiple transcription start sites were reported for some of these genes) [22]. As shown in Table 7, CoVote correctly identified 51 (78.5%) of these 65 known core promoter sequences. For 40 out of these 52 (76.9%) A. thaliana microRNA genes, CoVote predicted at least one core promoter region correctly. This analysis shows that our new promoter prediction method is fairly accurate. In comparison, TSSP (SoftBerry, http://www.softberry.com), which is one of the best promoter prediction methods for plants, only identified 39 (60%) promoters for 34 (65.4%) of these microRNA genes. Therefore, CoVote outperformed TSSP in this study.

**Table 7:**
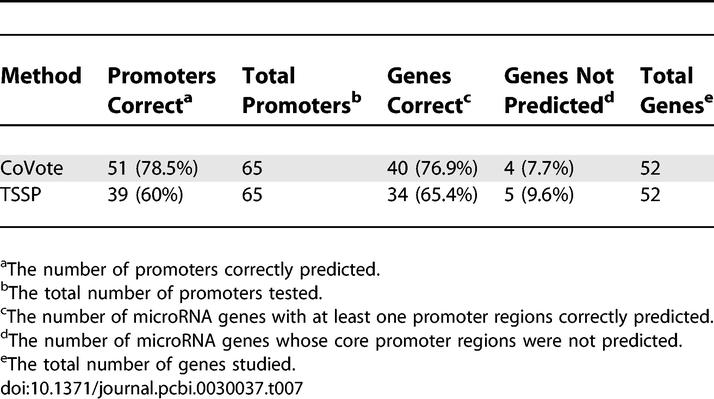
The Results of Promoter Prediction by CoVote and TSSP on A. thaliana MicroRNA Genes Whose Promoters Were Identified by Xie et al. [22]

Using a comparative genomics approach, Ohler et al. studied the flaking sequences of 43 pairs of orthologous C. elegans and C. briggsae pre-microRNAs, and reported *∼*250 bp conserved regions located around 200 bp upstream of the foldbacks [25]. In this study, we found that these conserved regions significantly overlapped with our predicted core promoter regions. In addition, the promoters of two microRNA genes in *H. sapiens, hsa-mir-23a∼27a∼24–2,* and *hsa-mir-371∼372∼373,* reported in [21,20], were also correctly predicted in our analysis.

The accuracy and false positive rate of CoVote were also assessed by known H. sapiens core promoters from DBTSS [32] (positive test set) and coding sequences (negative test set). The known core promoters of 4,189 H. sapiens protein-coding genes in the positive set were all correctly predicted. Ideally, we should evaluate false positive rates of these models with intergenic sequences that do not contain any promoters. However, it is difficult to obtain such intergenic sequences. Thus, we randomly chose 4,000 coding sequences as a negative control. For these, 4,000 negative test sequences, 1,325 (33.1%) were predicted to be core promoters, which gives the false positive rate of this method, although some of the predictions may be real.

### Significant Motifs in Core Promoters of MicroRNA Genes

To further characterize the predicted microRNA core promoters and gain a deep insight into microRNA transcriptional regulation, we performed a motif analysis to identify statistically significant and biologically meaningful motifs in the putative promoters. As shown in Figure 1, most putative promoters are located within the 500-bp upstream regions of pre-microRNA foldbacks. Therefore, for the microRNA genes that have multiple predicted promoter regions, we chose those promoters within the 500-bp upstream proximal regions of pre-microRNA hairpins for motif analysis. For those genes that do not have putative promoters within the 500-bp upstream regions, the promoters closest to the precursors were used.

In our study, we first applied two motif-finding algorithms, MEME [31] and WordSpy [26,27], to identify statistically overrepresented motifs. MEME is a statistical model–based algorithm for finding degenerate motifs, while WordSpy is a dictionary-based algorithm for finding a large number of exact motifs of high fidelity. We then conducted a whole-genome, Monte Carlo analysis to assess the biological relevance and specificity of the identified motifs to the core promoter regions of interest (see Motif Analysis). The motifs with *Z*-scores smaller than 3.0 were discarded, since they may also be prevalent in coding regions and/or other intergenic regions. The remaining ones are core promoter–specific motifs and likely to be biologically relevant to the transcriptional regulation of microRNA genes. Figure 2 lists some significant motifs that were identified by both motif-finding approaches and that were also reported in the literature as significant motifs in promoters of protein-coding genes. The whole list of motifs from WordSpy is given at http://cic.cs.wustl.edu/microrna/promoters.html. Many motifs from WordSpy match well with the motifs from MEME.

**Figure 2:**
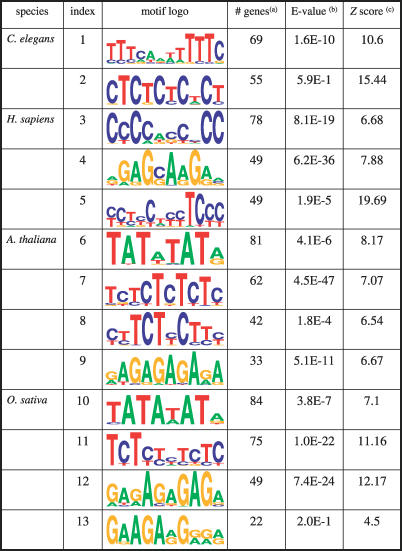
Significant Conserved Motifs Discovered in the Putative Promoters of the Four Species (A) The number of microRNA genes that contain the corresponding motifs in their upstream. (B) Expected frequencies of the corresponding motifs. (C) *Z*-scores obtained by Monte Carlo Simulations (see the section Motif Analysis).

In *C. elegans,* one of the most significant motifs identified by MEME has a consensus TTTCAATTTTTC (motif 1, Figure 2), which appears in 69 of the 73 predicted promoters. This motif matches the Inr (initiator) element, which has a weak consensus PyPyPyCANPyPyPyPyPy [23,24]. MEME also identified a significant motif in H. sapiens microRNAs that resembles the Inr element. This motif has a consensus CCCCACCTCC (motif 3, Figure 2), which appears in 78 putative promoters of H. sapiens microRNA genes. Wordspy also discovered several Inr-like motifs in both species.

TATA-box, which is one of the most well-known motifs in the core promoters of eukaryotic *class-II* genes, was discovered in A. thaliana and O. sativa (motifs 6 and 10, Figure 2). Among the 95 A. thaliana microRNA genes whose promoters were predicted by CoVote, 81 (85.3%) contain TATA-box. This observation is consistent with the experimental result in [22]. Specifically, Xie et al. reported that 42 (86.5%) of 52 A. thaliana microRNA genes contained TATA-box in their promoters [22]. In *O. sativa,* 84 of 114 (73.7%) microRNA genes contain TATA-box in their promoters. Although MEME did not report TATA-box in the promoters of C. elegans and H. sapiens microRNA genes, WordSpy identified it as a significant motif. We further scanned the putative promoters of C. elegans and H. sapiens microRNA genes with the TATA-box weight matrix curated in the Eukaryotic Promoter Database (EPD) (http://www.epd.isb-sib.ch). Including *hsa-mir-371∼372∼373,* whose promoter regions were analyzed by Houbaviy et al. [21], 35 (33%) of 107 H. sapiens microRNA genes and 34 (47%) of 73 C. elegans microRNA genes contain the canonical TATA-box in their promoters. The *Z*-scores of TATA-box in the promoters of microRNA genes in H. sapiens and C. elegans are 8.4 and 3.38, respectively, showing that TATA-box is a significant motif in the promoters of microRNA genes in these two species. Note that the frequency of TATA-box in plant microRNAs is nearly twice of that in animal microRNAs. This discrepancy deserves some further investigations.

Interestingly, CT-repeat microsatellites are significant motifs in the putative promoters of all four species (motifs 2, 4, 5, 7, 8, 9, 11, 12, and 13, Figure 2). To elucidate the significance of CT repeats in microRNA gene promoters, we performed several additional analyses. First, we analyzed the occurrences of CT repeats in the 2,000-bp upstream sequences of pre-microRNAs in all four species. As shown in Figure 3, in all four species tested, most microRNA genes have CT repeats in the 500-bp upstream regions of microRNA foldbacks. Second, we estimated the expected frequencies of CT repeats in the whole genomes of these species by a Monte Carlo simulation. Briefly, for each species, we randomly sampled *n* sequences with a length of 500 bp from its genome, where *n* was the number of microRNA genes whose upstream regions were analyzed for occurrences of CT repeats. Both strands of the genome sequences were scanned with the matrices of CT-repeat motifs listed in Figure 2 and other predefined CT-repeat sequences, including (CT)n, (CCT)n, (CTT)n, (CCTT)n, (CGCT)n, (CCTCG)n, (CCTCT)n, (CGTCT)n, and (CTCTT)n [33–36]. We then calculated the percentage of these sequences that contain CT repeats. We repeated the sampling 10,000 times, and computed the average percentage and the standard deviation of CT-repeat occurrences. As shown in Figure 3, in each of these four species the expected frequency in the whole genome is much lower than that in the promoter regions of microRNA genes. We also analyzed the distribution of CT repeats in the experimentally identified promoters of the 52 A. thaliana microRNA genes [22], and calculated the distances between the CT repeats and the TSSs. As shown in Table 8, 40 of these 52 genes contain CT repeats; in 30 of these 52 genes, the distances between CT repeats and TSSs are less than 100 bp. Additionally, the experimentally identified promoter regions of two H. sapiens microRNA genes, *hsa-mir-23a∼27a∼24–2* [20] and *hsa-mir-371∼372∼373* [21], contain CT repeats. The −56 to −34 upstream region of *has-mir-23a∼27a∼24–2* is CTCTCTCTCTCTTTCTCCCCTCC [20]. The −43 to −34 upstream region of *hsa-mir-371∼372∼373*, which is located closely nearby in the upstream of the reported TATA-box, contains a shorter CT repeat, CTCTCACCCT [21]. It has been shown that CT repeats are functional elements in the promoters of protein-coding genes in many mammalian species [37–40], Gallus gallus [41–43], and Drosophilia melanogaster [34,44,45]. Similar CT-repeat microsatellites in the core promoter regions of protein coding genes were also reported recently in A. thaliana and O. sativa [33,35,36]. Furthermore, initiator elements are pyrimidine-rich and contain CT repeats [45,42]. From a structure viewpoint, CT repeats can form non–B-DNA, which may potentially play important roles in gene transcription activation [46,47]. The frequent occurrence and the conservation across all four tested species suggest that CT repeats may play an important role in the transcription of microRNA genes.

**Figure 3:**
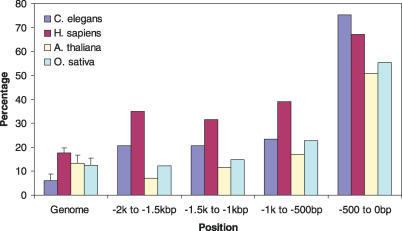
The Distributions of CT Repeats The first group to the left of the figure shows the distributions of CT repeats in the genomes of the four species studied, estimated by a Monte Carlo simulation. The subsequent groups show the distributions of CT repeats in the upstream of microRNA hairpins. The vertical axis is the percentage of microRNA genes and randomly sampled sequences that contain CT repeats (see text).

**Table 8:**
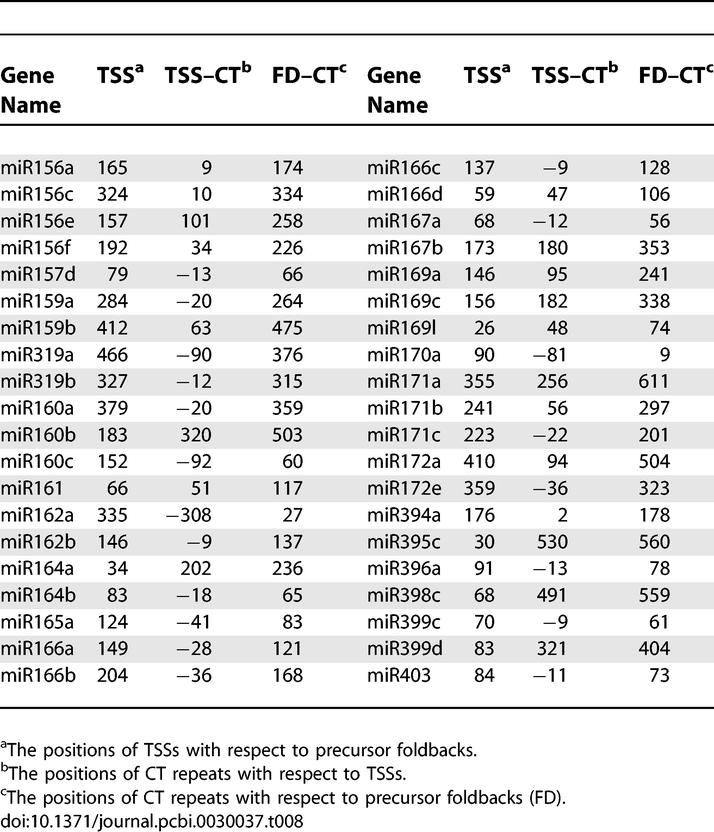
The Distances between CT Repeats and TSSs in the Promoters of 40 of 52 A. thaliana MicroRNA Genes Analyzed by Xie et al. [22]

A CpG island is one of the significant characteristics in the promoters of Eukaryotic *class-II* genes. We analyzed the presence of CpG islands in the upstream sequences of pre-microRNAs in all four species, as well as in the upstream sequences of 49 C. briggsae and 113 M. musculus microRNA genes. C. briggsae and M. musculus microRNA genes were included in order to form three pairs of evolutionarily closely related species, C. elegans versus *C. briggsae, H. sapiens* versus *M. musculus,* and A. thaliana versus *O. sativa,* for conservation analysis. We first identified CpG islands with CpGProD [48] and further confirmed the results with CpGPlot (http://bioweb.pasteur.fr/seqanal/interfaces/cpgplot.html). As shown in Table 9, a small number of microRNA genes in these species, except *A. thaliana,* have CpG islands in their upstream regions. The list of microRNA genes that contain CpG islands in their upstream sequences is given at http://cic.cs.wustl.edu/microrna/promoters.html. Two interesting observations are worth mentioning. First, CpG islands are often located close to pre-microRNA hairpins. Second, for most CpG-island–containing microRNA genes, their corresponding orthologous genes in closely related species also contain CpG islands in the upstream sequences. This may imply that CpG islands are evolutionarily conserved to a certain degree in these microRNA genes, and may be involved in the regulation of microRNA genes. However, none of the A. thaliana microRNA genes contain CpG islands, whereas 25 O. sativa microRNA genes do. It has been estimated that, in mammals, CpG islands are associated with approximately half of the promoters of protein coding genes [23]. CpG islands are frequently associated with ubiquitously expressed housekeeping genes [23]; thus, their roles in the regulation of those microRNA genes require further study.

**Table 9:**
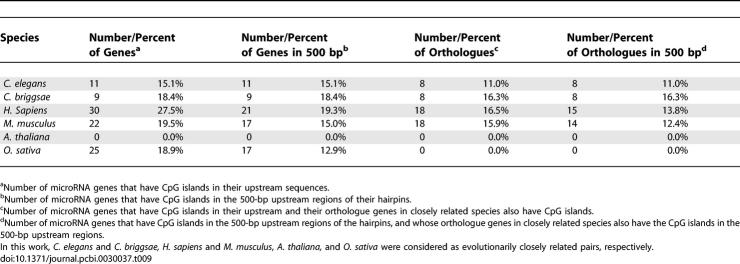
Putative CpG Islands in the Upstream Regions of Corresponding Hairpinned pre-MicroRNAs

Besides these conserved motifs, we also discovered several significant motifs that are specific to one of the four species studied. Two motifs (motifs 1 and 2, Figure 4) are specific to C. elegans microRNA genes, which match the consensus sequences of two motifs (CTCCGCCC and GCGTGGCS, S = C or G) conserved in the upstream of 43 pairs of C. elegans and C. briggsae orthologous microRNA genes [25]. A novel motif (motif 7, Figure 4) appears specifically in promoter regions of 61 A. thaliana microRNA genes. We further analyzed the distribution of this motif in the experimentally identified promoters of the 52 A. thaliana microRNA genes [22]: 24 promoters of 20 microRNA genes contain this motif. In most of these 24 promoters, the distance between this motif and TSS is smaller than 100 bp. Among four motifs that are specific to the promoters of O. sativa microRNA genes, motifs 9 and 10 in Figure 4 are known plant motifs reported in the literature. Motif 9 is an RY-repeat, which is conserved in the promoters of seed-specific genes in both monocot and dicot species [49–51]. Motif 10 has been found in the promoters of some anaerobic genes involved in the fermentative pathway of different plant species [52]. Motif 11 has been reported to be the binding site of HNF6 (Hepatocyte nuclear factor-6) in human and mouse by ChIP–chip experiments [53], while its function in plants remains unknown. There are two additional interesting observations on the motifs specific to O. sativa microRNA genes. First, all O. sativa motifs in Figure 4 have repetitive patterns in their consensus. Motif 8 has two copies of GCTA, motif 9 contains two copies of CATG, motif 10 can be viewed as CTG-repeats, and motif 11 has two copies of CGAT. Second, motifs 8, 9, and 11 are palindromic. Since palindromic patterns have been shown in binding sites of some transcription factors such as nuclear receptors in mammalian species [54], it may suggest that these three motifs are involved in the transcription of microRNA genes. In additional, four novel motifs discovered in the putative promoters of H. sapiens microRNA genes are all functionally unknown and need further study. Sequence similarities in promoters of *Arabidopsis*-specific microRNA genes have been addressed [55]. Therefore, although the functions of these species-specific motifs remain unclear, they will be important assets for future research, such as developing a new method for genome-wide identification of novel microRNA genes and conducting a wet lab microRNA analysis.

**Figure 4:**
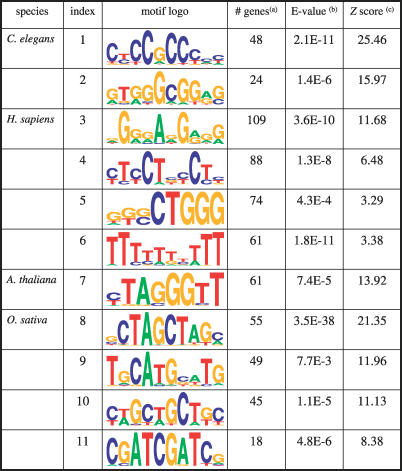
Significant Species-Specific Motifs Discovered in the Putative Promoters of MicroRNA Genes in Four Species. (A–C) The same as in Figure 2.

### Conclusions

In summary, we extensively analyzed the promoters of the known intergenic microRNA genes in four model species, *C. elegans, H. sapiens, A. thaliana,* and O. sativa. The genome-wide evidence from these four species showed that most, if not all, microRNA genes have the same type of promoters as protein-coding genes, and therefore are very likely to be transcribed by pol II. Our study extended the results on a small number of individual microRNA genes in H. sapiens [21,20] and A. thaliana [22] to all known microRNA genes in the four model species.

Moreover, with a new promoter identification method, we also located the core promoter regions of most known microRNA genes of these four species. The position distribution of putative promoters with respect to microRNA hairpins suggests that the core promoters of most microRNA genes are close to corresponding pre-microRNA hairpins (in the case of polycistronic microRNA genes, core promoters are close to the first pre-microRNA hairpins).

Furthermore, our extensive motif analysis of these putative promoters identified many *cis-*elements that are essential to the initiation of gene transcription. CT-repeat microsatellites were found to be conserved in all four species. Inr-like elements, which are relatively common in the promoters of protein-coding genes, were also discovered in the microRNA genes of C. elegans and H. sapiens. On the other hand, our results indicated that TATA-box does not seem to be necessary for most microRNA genes in C. elegans and *H. sapiens,* although most studied microRNA genes of A. thaliana and O. sativa contain TATA-box. Finally, CpG islands were discovered in a small portion of C. elegans and H. sapiens microRNA genes and their orthologues in C. briggsae and *M. musculus,* respectively. However, none of the A. thaliana microRNA genes contained CpG islands, although their O. sativa orthologues were found to contain CpG islands in their upstream sequences. Additionally, some motifs were discovered to be specific to individual species studied.

We expect our results on the putative promoters and the sequence motifs to be useful for future microRNA prediction and for elucidating the details of the regulation of microRNA gene transcription.

Additional supporting results and data files are available at http://cic.cs.wustl.edu/microrna/promoters.html.

## Abbreviations

CoVote: common query voting
Inr: initiator
pol II: RNA polymerase II
pol III: RNA polymerase III
SVM: support vector machine
TSS: transcription start site
